# Change in ankle-brachial index over time and mortality in diabetics with proteinuria 

**DOI:** 10.5414/CN107463

**Published:** 2012-07-12

**Authors:** Sirin Jiwakanon, Sharon Adler, Rajnish Mehrotra

**Affiliations:** 1 Hatyai Hospital, Hatyai, Songkhla, Thailand,; 2 Los Angeles Biomedical Research Institute at Harbor-UCLA, Torrance, and; 3 David Geffen School of Medicine at UCLA, Los Angeles, CA, USA

**Keywords:** all-cause mortality, ankle-brachial index, chronic kidney disease, peripheral arterial disease, proteinuria, Type 2 diabetes

## Abstract

Peripheral arterial disease is common in diabetic chronic kidney disease (CKD) and is characterized either by abnormally low or high ankle-brachial index (ABI). Whether low or high ABI carries similar prognostic value is unknown. The association of baseline ABI with all-cause mortality over 40 ± 21 months (mean ± SD) was ascertained in 167 proteinuric diabetics (age 57 ± 7 years; median urine protein-creatinine, 2.5 mg/mg). Association of change in ABI with all-cause mortality was determined in 75 subjects with normal ABI (0.9 – 1.3) at baseline. Among 167 participants, 41% had an abnormal ABI: < 0.9, 18%; and > 1.3 or non-compressible arteries, 23%. Only individuals with low ABI had a significantly higher risk for all-cause mortality (hazards ratio (95% confidence interval), HR: 2.23 (1.07, 4.65)). In subjects with normal ABI at baseline with follow-up measurement (n = 75), vascular disease worsened in 39% over 23 ± 6 months: 17% had either a decrease in ABI by ≥ 0.1 or a final ABI < 0.9, and 21% had a final ABI > 1.3 or non-compressible arteries. Only individuals who had a decrease in ABI over time had a significantly higher risk for death (adjusted HR, 7.41 (1.63, 33.65)). Peripheral arterial disease is not uncommon and progresses rapidly in individuals with diabetes and proteinuria. Low or declining ABI is a strong predictor of all-cause mortality. Routine measurement of ABI is a simple bed-side procedure that may permit easy risk-stratification in diabetic CKD patients.

## Introduction 

Diabetes is the most common cause of non-traumatic amputation in the United States; this higher risk is, at least in part, secondary to a higher prevalence and severity of peripheral arterial disease in diabetics [1, 2, 3]. The problem is further accentuated in diabetics with chronic kidney disease (CKD), and both a reduction in glomerular filtration rate and albuminuria are associated with a higher likelihood of peripheral arterial disease [4, 5, 6, 7]. Ankle-brachial index (ABI) is a readily obtainable measure at the bedside and occlusive peripheral arterial disease – invariably secondary to atherosclerosis – is characterized by low ABI (< 0.9). Low ABI is also a highly sensitive marker of systemic vascular disease burden and is a strong, independent predictor of fatal and non-fatal cardiovascular events in individuals with and without diabetes, with and without CKD [8, 9, 10, 11, 12, 13, 14]. However, many diabetics have high ABI (> 1.3 or 1.4) and a substantial proportion have non-compressible peripheral arteries [15, 16]. High ABI is considered to be a marker of vascular stiffness and/or medial artery calcification. However, some individuals with high ABI have evidence for underlying occlusive peripheral arterial disease [15]. There is a paucity of studies that have examined the relationship of high ABI to patient outcomes –   two studies have demonstrated that high ABI is associated with higher mortality in hemodialysis patients [17, 18]. To our knowledge, there are no such studies in patients with earlier stages of CKD. 

Studies from the general population suggest that decrease in ABI over time, in addition to low ABI at baseline, is associated with higher increased risk for non-fatal cardiovascular events or all-cause mortality [19, 20, 21]. There are no studies that have described the association of change in ABI over time with outcome in patients with CKD. Furthermore, many diabetics have non-compressible arteries leading to an elevated ABI [22]; whether increase in ABI over time is associated with a higher risk for death has not been tested even in individuals without CKD. Closing these gaps in our knowledge is critical to validate the use of repeat measurement of ABI for risk prediction in CKD. We undertook this study to test the following two hypotheses: in individuals with diabetes and proteinuria, 1) both low and high ABI are associated with a higher risk for death, and 2) both a decrease and an increase in ABI over time is associated with higher all-cause mortality. 

## Subjects and methods 

### Inclusion criteria and subject assessment 

This analysis is based upon data collected for subjects enrolled in a prospective cohort study of coronary artery calcification in individuals with Type 2 diabetes and diabetic nephropathy not undergoing maintenance dialysis. Data from this cohort has previously been published [23, 24]. Subjects were considered to have Type 2 diabetes if the diagnosis was made after the age of 30 and if they had been treated either with diet or oral medications for at least 6 months. The presence of diabetic nephropathy was defined as either the presence of consistent findings on a kidney biopsy, or a presumptive diagnosis using criteria similar to those used by the Family Investigation of Nephropathy in Diabetes (FIND) study: urine protein-creatinine ratio ≥ 0.5 mg/mg at least once within 12 months before enrollment and diabetes duration of either ≥ 10 years, or in individuals with diabetic retinopathy, diabetes duration of ≥ 5 years [25]. This study was approved by the Institutional Review Board of Los Angeles Biomedical Research Institute at Harbor-UCLA. 

Subjects were evaluated at the outpatient general clinical research center at Los Angeles Biomedical Research Institute after an overnight fast and underwent clinical and laboratory (urine and blood) assessment for ascertainment of the prevalence and/or severity of traditional, diabetes-related, and renal-related risk factors. History of cardiovascular disease was defined by the presence of one of the following: angina using the Rose questionnaire, history of myocardial infarction, or cerebrovascular accident, or previous revascularization. Electron-beam computed tomography (EBCT) scan was done at the baseline visit to measure the coronary artery calcification score. Estimated glomerular filtration rate (eGFR) was calculated using the abbreviated four-variable Modification of Diet in Renal Disease equation [26]. Serum albumin level was measured using bromcresol purple method. Serum intact parathyroid hormone (PTH) and homocysteine levels were measured using immunochemiluminometric assays (Quest Diagnostic Laboratories, San Juan Capistrano, CA, USA), and serum 25-hydroxy vitamin D was measured using liquid chromatography, tandem mass spectroscopy at Quest Diagnostic Laboratories (San Juan Capistrano, CA, USA). 

After the baseline assessment, subjects were invited for follow-up visits at 12 and 24 months. However, if the subjects required maintenance dialysis prior to these pre-determined intervals, all attempts were made to complete one follow-up assessment within 3 months of the first dialysis treatment. This analysis is based upon data collected at baseline and the last follow-up visit. 

### Measurement of ankle-brachial index (ABI) 

Doppler ultrasound (Nicolet Vascular Elite 100, Golden, CO, USA) with 8 MHz frequency probe was used for measuring systolic blood pressure in brachial, posterior tibial, and dorsalis pedis arteries bilaterally with the subject in the supine position. All ABI measurements were performed by three observers using the same equipment and a standardized protocol. Right and left ABI were calculated as the ratio of the highest systolic blood pressure in each of the two lower extremities (highest of posterior tibial or dorsalis pedis artery) to the brachial artery systolic blood pressure (highest of the right and left sides), respectively. Of the 170 subjects enrolled in the study, ABI were ascertained for 167 participants at baseline. Subjects were divided into three groups based upon this baseline measurement: (1) ABI < 0.9 in at least one of the two lower extremities, (2) ABI 0.9 – 1.3 in both lower extremities, and (3) ABI > 1.3 or non compressible artery in at least one lower extremity. 

Of the 99 subjects with normal ABI at baseline, follow-up assessment was completed for 75 individuals (76%). Reasons for inability to complete follow-up were: subject death, 7; subject refusal to come for follow-up visits, 10; lost-to-follow-up, 6; and ABI measurement not performed on follow-up visit, 1. There was no significant difference between any of the measured demographic, clinical, or laboratory characteristics of the included and excluded subjects. Individuals with normal baseline ABI were grouped into three categories based upon change in ABI over time as follows: (1) decrease in ABI ≥ 0.1 or final ABI < 0.9; (2) final ABI in the normal range (0.9 – 1.3); and (3) final ABI > 1.3 or non-compressible blood vessels. Annualized change in eGFR was determined for each subject; for the 6 individuals who had progressed to ESRD (8%) by the time of the last follow-up visit the final eGFR was assumed to be 10 ml/min/1.73 m^2^. 

### Ascertainment of subject survival 

Subjects were contacted once every 6 months via telephone to ascertain the vital status of each participant. If phone contact was unsuccessful, at least two certified letters were sent followed by home visit by a member of the study staff. To supplement the information obtained by study staff, a National Death Index screen was performed with follow-up through December 31, 2008. 

### Statistical analysis 

Data were summarized as mean and standard deviation, or median and interquartile range, or proportions. The significance of difference of characteristics at baseline between continuous variables was determined using unpaired t-test, Mann-Whitney rank-sum test, or 1-way analysis of variance, as appropriate. For categorical variables, the significance of difference was determined using the χ^2^-test. Time-to-event survival analysis among all 167 study participants was performed to determine the relationship of baseline ABI to patient survival. Subjects were censored either at the time of death, last phone contact by study staff, or last day of follow-up from National Death Index (12/31/2008), whichever was later. Log-rank test was used to determine the significance of difference in survival in the three study groups based upon baseline ABI. A Cox proportional hazards model was used to determine the baseline predictors of mortality among all 167 study participants. All variables listed in Table 1 were tested for their association with patient survival and for these analyses all continuous variables were divided into quartiles to avoid making the assumption that there was a linear association of the variable of interest with mortality. For adjusted analysis, variables significantly associated with survival on univariate analyses (p < 0.05) were entered into a forward selection multivariate model using likelihood ratios with the except of coronary artery calcification as it is the only variable that is not routinely measured in clinical practice. Similar univariate and multivariate analyses were performed to determine predictors of mortality in subjects with normal ABI at baseline in whom follow-up evaluation was completed. 

All statistical analyses were done using PASW Statistics 18.0 software (SPSS Inc, Chicago, IL, USA); p value < 0.05 was considered statistically significant. 

## Results 

### Patient characteristics 

The study cohort consisted of 167 Type 2 diabetics with proteinuria. At baseline, 41% had an abnormal ABI: 18% had ABI < 0.9, and 23% had either ABI > 1.3 or non-compressible arteries. Of the 38 subjects in the latter group, 8 had ABI > 1.3, and 30 had non-compressible arteries. The demographic, clinical, laboratory, and medication usage data, categorized by baseline ABI is summarized in Table 1. Compared to the two other groups, individuals with ABI < 0.9 were significantly more likely to have a previous history of cardiovascular disease, be current smokers, more likely to be prescribed beta blockers, and had higher coronary artery calcification scores (Table 1). In contrast, individuals with normal ABI at baseline were less likely to have hypertension, had higher diastolic blood pressure, and the lowest coronary artery calcification scores. Finally, individuals with ABI > 1.3 or non-compressible arteries had significantly higher serum creatinine and albuminuria at baseline compared to the other two groups. 

### Predictors of change in ABI over time 

Follow-up assessment was completed for 75 of the 99 subjects with normal ABI at baseline after a mean interval of 23 ± 6 months. There was no significant difference between any of the measured demographic, clinical, or laboratory characteristics of the included and excluded subjects. Vascular disease worsened over this follow-up period in 39% of subjects. 13 subjects (17%) had a final ABI < 0.9 (n = 8, mean decrease 0.2 ± 0.1, range 0.04 – 0.34), or had a decrease in ABI by at least 0.1 but still within the normal range (n = 5). Furthermore, 16 (21%) subjects had a final ABI > 1.3 (n = 11), or had non-compressible arteries (n = 5). The baseline characteristics, and change in renal function based upon time course of ABI is summarized in Table 2. Individuals with decrease in ABI by at least 0.1 or final ABI < 0.9 were substantially more likely to have a history of cardiovascular disease (Table 2). There was no significant difference in any of the other baseline characteristics, or medication usage between any of the three groups. 

### Association of baseline and change in ABI over time with subject survival 

**Figure 1.**
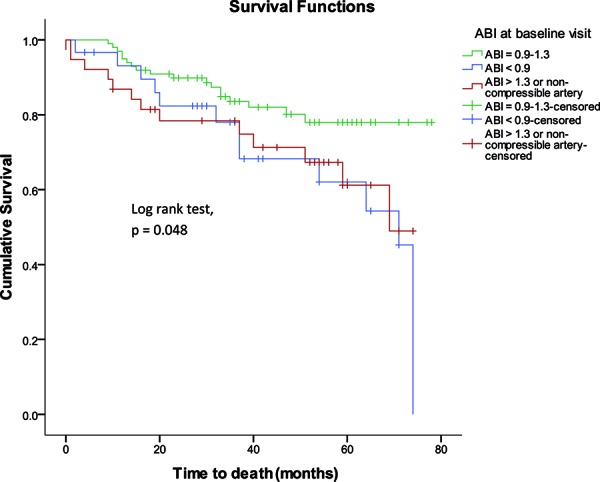
Kaplan-Meier plot showing the relationship of baseline ankle-brachial index to survival 167 subjects with Type 2 diabetes and proteinuria; Log rank p = 0.048.

Over a follow-up period of 40 ± 21 months, 43 of the 167 subjects evaluated at baseline died: ABI 0.9 – 1.3, 18 of 99 subjects (18%); ABI < 0.9, 12 of 30 (40%); and ABI > 1.3 or non-compressible, 13 of 38 (34%). Using time-to-event analysis, the risk for death was significantly higher in individuals with baseline ABI < 0.9 (hazards ratio (95% confidence interval): 2.23 (1.07, 4.65)); the higher risk for death in individuals with ABI > 1.3 or non-compressible arteries did not reach statistical significance (1.98 (0.97, 4.04)) (Table 3) (Figure 1). Other significant predictors of survival, on univariate analyses, are also summarized in Table 3. The qualitative trend for a higher risk for death with ABI < 0.9 persisted on multivariate analyses but did not reach statistical significance (adjusted HR, 1.79 (0.84, 3.84)). Race/ethnicity and baseline eGFR were identified as predictors of mortality in this cohort. Similar results were obtained when age, hypertension, and proteinuria were forced into the model or when eGFR was used as a continuous, rather than a categorical variable. 

**Figure 2.**
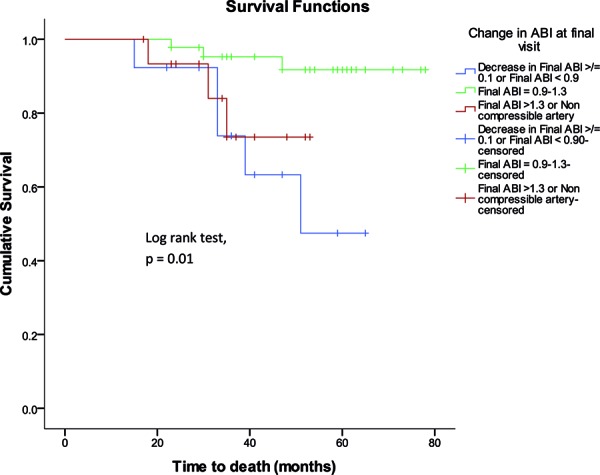
Kaplan-Meier plot showing the relationship of change in ankle-brachial index over time to survival probability in 75 subjects with normal ankle-brachial index over time; Log rank p = 0.01.

75 subjects with normal ABI at baseline had repeat evaluation after a mean interval of 23 months. Over a mean follow-up period of 21 ± 16 months from the second assessment (follow-up from baseline assessment, 44 ± 17 months), 11 subjects (15%) died. This included 5 of the 13 subjects with a decrease in ABI over time (39%), 3 of the 16 with increase in ABI over time (19%), and 3 of the 46 with ABI that stayed within the normal range (7%). The risk for death was significantly higher in subjects who had a decrease in ABI over time (Figure 2) (Table 4). Upon adjusting the data for race/ethnicity, a final ABI < 0.9, or decrease in ABI by at least 0.1 remained a significant independent predictor of mortality (adjusted HR, 7.41 (1.63, 33.65)). Similar results were obtained when age, hypertension, and proteinuria were forced into the model as additional covariates. 

## Discussion 

To our knowledge, this is the first study to report on the progression of peripheral arterial disease in diabetics with CKD and it allows us to make several important observations. First, over 40% of our study cohort of individuals with diabetes and proteinuria with an average eGFR of 58 ml/min/1.73 m^2^ had an abnormal ABI at baseline – the prevalence of high ABI or non-compressible arteries was somewhat more common than a low ABI. Second, almost 40% of subjects with normal ABI at baseline had worsening of vascular disease over a relatively short follow-up period of 23 months. Finally, both a low ABI at baseline or a decrease over time, were stronger predictors of mortality than a high baseline ABI or an increase over time. 

It has been estimated that the prevalence of ABI < 0.9 and > 1.3 in non-institutionalized American adults (≥ 40 years of age) with creatinine clearance > 60 ml/min/1.73 m^2^ is 3.7% and 3.4%, respectively [27]. Several studies have demonstrated a substantially higher prevalence of either a low or a high ABI in subjects with CKD, particularly in those with diabetes mellitus [9, 13, 16, 28]. The prevalence rate of abnormal ABI at baseline in our population is consistent with these previous findings. It is widely accepted that a low ABI is secondary to occlusive atherosclerosis in peripheral arteries and is strongly associated with distal ischemia and limb loss in the general population [29]. Furthermore, it is a marker of systemic atherosclerosis and in the general population has been associated with higher risk for fatal and non-fatal cardiovascular outcomes [30, 31, 32, 33, 34, 35]. Similar association of low ABI with cardiovascular events has been reported in populations with CKD [7, 13, 36]. Furthermore, in our study, individuals with low ABI were significantly more likely to have clinically manifest cardiovascular disease than the other two groups. This suggests that like in the general population, low ABI represents underlying atherosclerotic disease in individuals with CKD, a condition that is invariably associated with intimal calcification [37]. However, less is known about the pathophysiologic basis for the high prevalence of increased ABI in diabetic CKD subjects. It is likely that high ABI is a marker of increased vascular stiffness, with/without associated medial artery calcification – both of which are increased in prevalence and severity in diabetic CKD [16, 38, 39, 40]. Consistent with these considerations, individuals with either low or high ABI in our study had higher coronary artery calcification scores than those with normal ABI. It is possible that the calcification in the former group is largely intimal while in the latter is medial. However, this was not directly studied herein and remains speculative. 

There are no published studies that have reported serial measurements of ABI in any population of diabetic individuals with CKD or among those undergoing maintenance dialysis. Our study allows us to describe for the first time the natural history of ABI in a well-delineated cohort of individuals with diabetes and proteinuria. Over a relatively short average follow-up period of 23 months, the ABI of almost 40% of individuals with a normal value at baseline became abnormal. The incidence of an abnormal ABI in our cohort is considerably higher than reported in other high-risk populations. While 17% of our cohort had a clinically relevant decrease in ABI over 23 months, only 9.5% of elderly individuals enrolled in the Cardiovascular Health Study had a similar change (ABI decrease > 0.15 or final ABI < 0.9) over 6 years [41]. To our knowledge, there are no studies that have described the incidence of increase in ABI over time in individuals with or without CKD. In the cohort described herein, 1 in 5 of individuals with diabetes and proteinuria with normal ABI at baseline had a significant increase in ABI in less than 2 years. Individuals who had a significant decline in ABI over time were more likely to have a previous history of cardiovascular disease than the other two groups. There were no other demographic, clinical, or laboratory parameters at baseline that could allow us to predict which individuals would have worsening of peripheral arterial disease over time. This issue needs to be investigated further in future studies to allow us to develop interventions to slow the rapid progression of peripheral arterial disease in this high-risk population of individuals with diabetes and proteinuria. 

In addition to the first description of the natural course of subclinical peripheral arterial disease in individuals with diabetes and proteinuria, our study suggests that ABI may be a useful tool to risk stratify a high-risk CKD population. A low ABI at baseline was associated with a significantly higher risk for all-cause mortality; the trend for association of high ABI did not reach statistical significance. Low ABI has been demonstrated to be strongly predictive of all-cause and cardiovascular mortality in a variety of populations without kidney disease [8, 14, 30, 35, 42, 43]. Similar associations have been described in individuals with different stages of CKD, including those with end-stage renal disease undergoing hemodialysis [9, 13, 17, 18]. Our study corroborates the findings of these previous studies; the magnitude of increase in death risk with low ABI was similar to that seen with high serum C-reactive protein. There are substantially fewer studies that have examined the association of high ABI with hard outcomes – some such studies have demonstrated a higher risk for death in individuals with high ABI without kidney disease or among those undergoing hemodialysis [8, 17, 18]. To our knowledge, this study in individuals with diabetes and proteinuria is the first such study in CKD individuals not undergoing dialysis. The trend for higher risk for death in individuals with high ABI at baseline did not reach statistical significance. Given the relatively modest sample size, we cannot definitively exclude a higher risk in individuals with high ABI compared to those with normal assessment at baseline. However, it seems appropriate to conclude that a low ABI portends a significantly worse prognosis than either a high or normal ABI at baseline. 

Longitudinal assessment and risk prediction with change over time in this study cohort is perhaps the strongest argument in favor of further evaluating ABI for risk prediction in diabetic CKD. Consistent with our findings using the baseline measurement, it was a decline in ABI over time in individuals with previously normal ABI that was associated with the highest risk for all-cause mortality. These findings are consistent with observations of a higher death risk with decrease in ABI in individuals with known peripheral arterial disease [19, 20, 21]. However, there are no such studies in unselected population of individuals with or without kidney disease and our study is the first such demonstration. Moreover, we are not aware of any studies that have examined the risk associated with an increase in ABI over time in any population. In our study of diabetic CKD, the trend for a higher risk with increase in ABI over time did not reach statistical significance. Just like for the baseline assessment, we cannot definitively exclude that an increase in ABI identifies an individual with a higher risk for subsequent all-cause mortality. However, it appears reasonable to conclude that a decrease in ABI over time portends a substantially worse prognosis in individuals with previously normal ABI. It is probable that decline in ABI identifies individuals with the greatest worsening in systemic atherosclerosis which, in turn, is responsible for the higher all-cause mortality. 

The results of our study should be interpreted in light of its limitations. First, our study population was limited to individuals with diabetes and proteinuria with early stage CKD. Whether the predictive value of ABI and its change over time applies to individuals with diabetic end-stage renal disease or non-diabetics with kidney disease needs investigation in future studies. Second, the number of mortal events was relatively modest and this may have limited the statistical power for some of our analysis. This appears to particularly be the case for individuals with high or increasing ABI. Third, data on symptoms attributable to peripheral arterial disease (claudication or rest pain) were not available. There is concern that increased vascular stiffness may lead to an apparently normal or high ABI and thus, mask the presence of underlying occlusive vascular disease [15, 44]. This may be particularly true in individuals with diabetes mellitus and CKD who are more likely to develop medial artery calcification. Furthermore, the follow-up was not long enough for us to determine association of ABI with non-traumatic amputation. Fourth, we did not have access to information regarding cause of death and hence, we could not analyze the data for cause-specific mortality. Finally, our study cohort was comprised predominantly of Latinos; care should be exercised when extrapolating our findings to other racial/ethnic groups. Similarly, our findings of differential risk for different racial/ethnic groups should be interpreted with caution. 

In summary, in this analysis from a longitudinal prospective cohort, we report a very high prevalence of abnormal ABI in individuals with diabetes and proteinuria. In a significant proportion of individuals with normal ABI at baseline, it became abnormal over a relatively short period of time. Furthermore, low or declining ABI in individuals with diabetes and proteinuria is associated with 2.2- and 7.1-fold higher risk, respectively, for all-cause mortality. These findings provide strong support for using longitudinal assessment of ABI in the day-to-day assessment of individuals with diabetic CKD. Given that ABI is readily obtainable at the bedside at a substantially lower cost than other measures that have been shown to be associated with mortality in this population (viz., coronary artery calcification scores, measures of inflammation, or mineral metabolism) makes it even more attractive for routine clinical use. Future studies should investigate whether the risk associated with low or decreasing ABI at baseline is modifiable. 

## Acknowledgments 

This work was supported by grants from the National Institute of Health to Rajnish Mehrotra (RR18298) and to the General Clinical Research Center at Los Angeles Biomedical Research Institute (MO1-RR00425). 

## Potential conflicts of interest 

Rajnish Mehrotra and Sharon Adler are supported by a grant from DaVita Inc. Rajnish Mehrotra has received grant support and/or honoraria from Amgen, Baxter Healthcare, DaVita, Genzyme, Mitsubishi, Shire, Takeda, and Vifor. 

**Table 1.** Characteristic of 167 patients categorized by the baseline ankle-brachial index (ABI).

	ABI category				
	< 0.9	0.9 – 1.3	> 1.3 or non compressible	p value	Entire cohort
Subject number, n (%)	30 (18)	99 (59)	38 (23)		167
Demographics					
Age, years	60 ± 7	56 ± 7	58 ± 8	0.09	57 ± 7
Men, n (%)	15 (50)	53 (54)	23 (61)	0.66	91 (55)
Race/ethnicity, n (%)				0.66	
Latino	20 (67)	74 (75)	30 (79)		124 (74)
Non-Latino Whites	7 (23)	12 (12)	5 (13)		24 (14)
Non-Latino Blacks	2 (7)	9 (9)	3 (8)		14 (8)
Others	1 (3)	4 (4)	0		5 (3)
Clinical characteristics					
Diabetes duration, years	13 ± 6	15 ± 6	17 ± 6	0.07	15 ± 6
Hypertension, n (%)	27 (90)	76 (77)	35 (92)	0.05	138 (83)
History of cardiovascular disease, n (%)	17 (57)	29 (30)	8 (21)	0.01	54 (32)
Current smoker, n (%)	9 (30)	17 (17)	1 (3)	0.01	27 (16)
Past smoker, n (%)^c^	12 (52)	43 (51)	17 (49)	0.08	72 (50)
Body mass index, kg/m^2a^	29 (16)	29 (10)	29 (10)	0.97	29 (9)
Systolic blood pressure, mmHg	149 ± 24	157 ± 28	153 ± 28	0.33	155 ± 27
Diastolic blood pressure, mmHg	73 ± 10	79 ± 14	75 ± 10	0.04	77 ± 13
Pulse pressure, mmHg	76 ± 19	78 ± 23	79 ± 25	0.87	78 ± 23
Laboratory data					
Serum creatinine, mg/dl	1.3 ± 0.5	1.3 ± 0.5	1.6 ± 0.6	0.04	1.4 ± 0.5
Estimated glomerular filtration rate, ml/min/1.73 m^2^	58 ± 22	61 ± 24	51 ± 22	0.08	58 ± 23
Serum glucose, mg/dl^a^	155 (85)	147 (90)	159 (99)	0.95	154 (89)
Hemoglobin A_1_C, %	8.9 ± 2.4	8.5 ± 2.1	8.8 ± 2.2	0.62	8.6 ±2.2
Total cholesterol, mg/dl	186 ± 51	197 ± 53	181 ± 49	0.25	191 ± 52
Low-density lipoprotein cholesterol, mg/dl	106 ± 42	115 ± 45	103 ± 33	0.29	110 ± 42
Triglycerides, mg/dl^a^	180 (159)	150 (128)	144 (130)	0.68	151 (127)
Corrected serum calcium, mg/dl	9.9 ± 0.4	9.7 ± 0.4	9.8 ± 0.4	0.18	9.8 ± 0.4
Serum phosphorus, mg/dl	4.3 ± 0.6	4.2 ± 0.7	4.5 ± 0.7	0.20	4.3 ± 0.7
Serum albumin, g/dl	3.3 ± 0.6	3.3 ± 0.5	3.1 ± 0.6	0.10	3.2 ± 0.5
Serum parathyroid hormone, pg/ml^a^	45 (37)	41 (39)	58 (57)	0.10	45 (44)
Serum homocysteine, µmol/l^a^	13 (8)	12 (5)	15 (6)	0.06	13 (8)
Serum 25-hydroxy vitamin D, ng/ml	25 ± 10	22 ± 11	20 ± 10	0.12	22 ± 10
C-reactive protein > 0.4 mg/dl, n (%)	14 (47)	41 (41)	22 (58)	0.22	77 (46)
Urine protein-creatinine ratio, mg/mg^a^	2.0 (3)	2.2 (3)	4.3 (6)	0.06	2.5 (4)
Urine albumin-creatinine ratio, mg/mg^a^	1.7 (2)	1.7 (3)	3.3 (4)	0.03	1.9 (3)
Baseline medical therapy^b^					
ACEIs or ARBs, n (%)	24 (83)	74 (82)	33 (87)	0.81	131 (83)
β-blocker, n (%)	21 (72)	41 (46)	22 (58)	0.03	84 (54)
Acetylic salicylic acid and anti-platelet agents, n (%)	18 (62)	47 (52)	17 (45)	0.37	82 (52)
Diuretic, n (%)	16 (55)	47 (52)	24 (63)	0.52	87 (55)
Lipid-lowering agents, n (%)	22 (76)	62 (69)	28 (74)	0.72	112 (71)
Phosphate binders, n (%)	2 (7)	7 (7)	5 (13)	0.51	14 (9)
Vitamin D analogs, n (%)	0	1 (1)	1 (3)	0.59	2 (1)
Coronary artery calcium score^a^	258 (304)	105 (220)	169 (254)	0.001	135 (265)

Values expressed as mean ± standard deviation or percentage. ABI = ankle-brachial index; ACEI = angiotensin-converting enzyme inhibitors; ARBs = Angiotensin II receptor blockers. ^a^Values expressed as median and inter-quartile range; ^b^Data missing for 10 subjects; ^c^Data missing for 24 subjects.

**Table 2.** Baseline characteristics of 75 subjects with normal ankle-brachial index at baseline categorized by change in ankle-brachial index over time.

	Decrease ABI ≥ 0.10 or final ABI < 0.9	Final ABI 0.9 – 1.3	Final ABI > 1.3 or non compressible	p value
Subject number, n (%)	13 (17)	46 (61)	16 (21)	
Demographics				
Age, years	57 ± 6	56 ± 8	59 ± 7	0.60
Men, n (%)	5 (39)	24 (52)	12 (75)	0.13
Race/ethnicity, n (%)				0.86
Latino	8 (62)	36 (78)	10 (63)	
Non-Latino Whites	2 (15)	5 (11)	3 (19)	
Non-Latino Blacks	2 (15)	3 (7)	2 (13)	
Others	1 (8)	2 (4)	1 (6)	
Clinical characteristics				
Diabetes duration, years	14 ± 5	15 ± 6	16 ± 9	0.66
Hypertension, n (%)	11 (85)	33 (72)	14 (88)	0.34
History of cardiovascular disease, n (%)	8 (62)	7 (15)	5 (31)	< 0.01
Current smoker, n (%)	2 (15)	8 (17)	5 (31)	0.44
Past smoker, n (%)^c^	5 (42)	17 (44)	8 (67)	0.34
Body mass index, kg/m^2a^	32 (8)	29 (10)	29 (13)	0.35
Systolic blood pressure, mmHg	161 ± 22	156 ± 31	159 ± 22	0.81
Diastolic blood pressure, mmHg	80 ± 12	79 ± 15	77 ± 14	0.78
Pulse pressure, mmHg	80 ± 20	77 ± 24	83 ± 21	0.65
Laboratory data				
Serum creatinine, mg/dl	1.4 ± 0.8	1.3 ± 0.5	1.4 ± 0.5	0.69
Estimated glomerular filtration rate, ml/min/1.73 m^2^	60 ± 30	60 ± 23	61 ± 23	1.00
Change in estimated glomerular filtration rate, ml/min/1.73 m^2^/year^a^	–8 (10)	–7 (11)	–9 (13)	0.75
Serum glucose, mg/dl^a^	144 (77)	167 (101)	126 (87)	0.09
Hemoglobin A_1_C, %	8.5 ± 1.8	8.6 ± 2	8.3 ± 2.2	0.87
Total cholesterol, mg/dl	212 ± 65	198 ± 50	179 ± 38	0.22
Low-density lipoprotein cholesterol, mg/dl	125 ± 51	114 ± 42	104 ± 31	0.41
Triglycerides, mg/dl^a^	132 (171)	173 (109)	117 (81)	0.11
Corrected serum calcium, mg/dl	9.7 ± 0.5	9.7 ± 0.4	9.7 ± 0.4	0.84
Serum phosphorus, mg/dl	4.4 ± 0.6	4.2 ± 0.7	4.1 ± 0.6	0.57
Serum albumin, g/dl	3.3 ± 0.6	3.3 ± 0.6	3.3 ± 0.3	0.86
Serum parathyroid hormone, pg/ml^a^	55 (75)	40 (37)	39 (39)	0.41
Serum homocysteine, µmol/l^a^	12 (6)	13 (6)	12 (5)	0.89
Serum 25-hydroxy vitamin D, ng/ml	19 ± 10	22 ± 11	22 ± 11	0.63
C-reactive protein > 0.4 mg/dl, n (%)	7 (54)	17 (37)	7 (44)	0.54
Urine protein-creatinine ratio, mg/mg^a^	1.8 (1)	2.2 (3)	2.5 (4)	0.75
Urine albumin-creatinine ratio, mg/mg^a^	1.3 (1)	1.8 (3)	2.0 (3)	0.65
Baseline medical therapy^b^				
ACEIs or ARBs, n (%)	11 (92)	35 (81)	12 (80)	0.67
β Blocker, n (%)	7 (58)	15 (35)	7 (47)	0.31
Acetylic salicylic acid and anti-platelet agents, n (%)	4 (33)	25 (58)	9 (60)	0.28
Diuretic, n (%)	7 (58)	19 (44)	10 (67)	0.28
Lipid-lowering agents, n (%)	10 (83)	27 (63)	11 (73)	0.36
Phosphate binders, n (%)	3 (25)	3 (7)	1 (7)	0.15
Coronary artery calcium score^a^	130 (162)	89 (282)	108 (125)	0.60

Values expressed as mean ± standard deviation or percentage. ABI = ankle-brachial index; ACEI = angiotensin-converting enzyme inhibitors; ARBs = Angiotensin II receptor blockers. ^a^Values expressed as median and inter-quartile range, ^b^Data missing for 5 subjects, ^c^Data missing for 12 subjects.

**Table 3.** Baseline significant predictors of mortality in 167 subjects using the Cox proportional hazards model.

Variables	Univariate		Multivariate	
	Hazard ratio (95% CI)	p value	Hazard ratio (95% CI)	p value
Male gender (Ref: female)	1.88 (1.00, 3.53)	0.05		
Race/ethnicity (Ref: Latino)		0.003		0.001
Non-Latino Whites	3.07 (1.53, 1.16)	0.002	3.08 (1.51, 6.27)	0.002
Non-Latino Blacks	3.99 (1.47, 10.84)	0.007	5.40 (1.92, 15.18)	0.001
Estimated GFR (Ref: 4^th^ quartile (75.8 – 117.3 ml/min/1.73 m^2^))		0.03		0.01
3^rd^ quartile (54.3 – 75 ml/min/1.73 m^2^)	0.44 (0.16, 1.25)	0.12	0.41 (0.15, 1.15)	0.10
2^nd^ quartile (39.7 – 54 ml/min/1.73 m^2^)	0.90 (0.37, 2.23)	0.82	0.71 (0.28, 1.79)	0.46
1^st^ quartile (14.4 – 39 ml/min/1.73 m^2^)	1.77 (0.82, 3.85)	0.15	1.80 (0.83, 3.91)	0.14
C-reactive protein > 0.4 mg/dl	1.97 (1.07, 3.63)	0.03		
Coronary artery calcium score (Ref: 1^st^ quartile: 0 – 17)		0.004		
2^nd^ quartile (18 – 135)	2.58 (0.79, 8.44)	0.12		
3^rd^ quartile (140 – 273)	6.14 (2.07, 18.19)	0.001		
4^th^ quartile (283 – 977)	3.01 (0.96, 9.46)	0.06		
Baseline ABI (Ref: 0.9 – 1.3)		0.06		
ABI < 0.9	2.23 (1.07, 4.65)	0.03		
ABI > 1.3 or non-compressible	1.98 (0.97, 4.04)	0.06		

The adjusted model was built using forward selection of variables significant on univariate analysis (except coronary artery calcification score). GFR = glomerular filtration rate; ABI = ankle-brachial index.

**Table 4.** Predictors of mortality for 75 subjects with normal ankle brachial index at baseline and repeat evaluation after a mean interval of 23 months using the Cox proportional hazard model.

	Univariate		Multivariate	
Variables	Hazard ratio (95% CI)	p value	Hazard ratio (95% CI)	p value
Race/ethnicity (Ref: Latino)^a^		0.004		0.005
Non-Latino Whites	18.14 (3.43, 95.94)	0.001	17.13 (2.95, 99.44)	0.002
Non-Latino Blacks	25.12 (3.32, 190.29)	0.002	35.81 (3.95, 325.05)	0.001
Coronary artery calcium score > 112 (Ref: < 112)	4.65 (1.00, 21.54)	0.05		
Final ABI (ref.: 0.9 – 1.3)^b^		0.03		0.04
Decrease by ≥ 0.1 or final ABI < 0.9	7.05 (1.68, 29.58)	0.008	7.41 (1.63, 33.65)	0.01
Final ABI > 1.3 or non-compressible	4.32 (0.86, 21.63)	0.08	3.64 (0.67, 19.79)	0.14

For multivariate analysis, ^a^adjusted for change in final ABI over time, ^b^adjusted for race/ethnicity-model built using forward selection of variables significant on univariate analysis (except coronary artery calcification score). ABI = ankle-brachial index.

## References

[1] Melton LJ Macken KM Palumbo PJ Elveback LR Incidence and prevalence of clinical peripheral vascular disease in a population-based cohort of diabetic patients. Diabetes Care. 1980; 3: 650–654. 7449595 10.2337/diacare.3.6.650

[2] Welborn TA Knuiman M McCann V Stanton K Constable IJ Clinical macrovascular disease in Caucasoid diabetic subjects: logistic regression analysis of risk variables. Diabetologia. 1984; 27: 568–573. 6530052 10.1007/BF00276969

[3] Walters DP Gatling W Mullee MA Hill RD The prevalence, detection, and epidemiological correlates of peripheral vascular disease: a comparison of diabetic and non-diabetic subjects in an English community. Diabet Med. 1992; 9: 710–715. 1395462 10.1111/j.1464-5491.1992.tb01878.x

[4] Jager A Kostense PJ Ruhé HG Heine RJ Nijpels G Dekker JM Bouter LM Stehouwer CD Microalbuminuria and peripheral arterial disease are independent predictors of cardiovascular and all-cause mortality, especially among hypertensive subjects: five-year follow-up of the Hoorn Study. Arterioscler Thromb Vasc Biol. 1999; 19: 617–624. 10073965 10.1161/01.atv.19.3.617

[5] Selvin E Köttgen A Coresh J Kidney function estimated from serum creatinine and cystatin C and peripheral arterial disease in NHANES 1999-2002. Eur Heart J. 2009; 30: 1918–1925. 19487236 10.1093/eurheartj/ehp195 PMC2719699

[6] Choi SW Yun WJ Kim HY Lee YH Kweon SS Rhee JA Choi JS Shin MH Association between albuminuria, carotid atherosclerosis, arterial stiffness, and peripheral arterial disease in Korean type 2 diabetic patients. Kidney Blood Press Res. 2010; 33: 111–118. 20431302 10.1159/000313594

[7] Luo Y Li X Li J Wang X Xu Y Qiao Y Hu D Ma Y Peripheral arterial disease, chronic kidney disease, and mortality: the Chinese Ankle Brachial Index Cohort Study. Vasc Med. 2010; 15: 107–112. 20133341 10.1177/1358863X09357230

[8] Resnick HE Lindsay RS McDermott MM Devereux RB Jones KL Fabsitz RR Howard BV Relationship of high and low ankle brachial index to all-cause and cardiovascular disease mortality: the Strong Heart Study. Circulation. 2004; 109: 733–739. 14970108 10.1161/01.CIR.0000112642.63927.54

[9] Guerrero A Montes R Muñoz-Terol J Gil-Peralta A Toro J Naranjo M González-Pérez P Martín-Herrera C Ruiz-Fernández A Peripheral arterial disease in patients with stages IV and V chronic renal failure. Nephrol Dial Transplant. 2006; 21: 3525–3531. 16940320 10.1093/ndt/gfl470

[10] Li J Luo Y Xu Y Yang J Zheng L Hasimu B Yu J Hu D Risk factors of peripheral arterial disease and relationship between low ankle - brachial index and mortality from all-cause and cardiovascular disease in Chinese patients with type 2 diabetes. Circ J. 2007; 71: 377–381. 17322639 10.1253/circj.71.377

[11] Liew YP Bartholomew JR Demirjian S Michaels J Schreiber MJ Combined effect of chronic kidney disease and peripheral arterial disease on all-cause mortality in a high-risk population. Clin J Am Soc Nephrol. 2008; 3: 1084-1089. 18337552 10.2215/CJN.04411007 PMC2440260

[12] Bundó M Muñoz L Pérez C Montero JJ Montellà N Torán P Pera G Asymptomatic peripheral arterial disease in type 2 diabetes patients: a 10-year follow-up study of the utility of the ankle brachial index as a prognostic marker of cardiovascular disease. Ann Vasc Surg. 2010; 24: 985–993. 21035692 10.1016/j.avsg.2010.06.001

[13] Chen SC Chang JM Hwang SJ Tsai JC Liu WC Wang CS Lin TH Su HM Chen HC Ankle brachial index as a predictor for mortality in patients with chronic kidney disease and undergoing haemodialysis. Nephrology (Carlton). 2010; 15: 294–299. 20470297 10.1111/j.1440-1797.2010.01187.x

[14] Quiles J Morillas P Bertomeu V Mazon P Cordero A Soria F Combination of ankle brachial index and diabetes mellitus to predict cardiovascular events and mortality after an acute coronary syndrome. Int J Cardiol. 2011; 151: 84-88.; 10.1016/j.ijcard.2010.04.097 20488566

[15] Aboyans V Ho E Denenberg JO Ho LA Natarajan L Criqui MH The association between elevated ankle systolic pressures and peripheral occlusive arterial disease in diabetic and nondiabetic subjects. J Vasc Surg. 2008; 48: 1197–1203. 18692981 10.1016/j.jvs.2008.06.005

[16] Leskinen Y Salenius JP Lehtimäki T Huhtala H Saha H The prevalence of peripheral arterial disease and medial arterial calcification in patients with chronic renal failure: requirements for diagnostics. Am J Kidney Dis. 2002; 40: 472–479. 12200797 10.1053/ajkd.2002.34885

[17] Ono K Tsuchida A Kawai H Matsuo H Wakamatsu R Maezawa A Yano S Kawada T Nojima Y Ankle-brachial blood pressure index predicts all-cause and cardiovascular mortality in hemodialysis patients. J Am Soc Nephrol. 2003; 14: 1591–1598. 12761260 10.1097/01.asn.0000065547.98258.3d

[18] Kitahara T Ono K Tsuchida A Kawai H Shinohara M Ishii Y Koyanagi H Noguchi T Matsumoto T Sekihara T Watanabe Y Kanai H Ishida H Nojima Y Impact of brachial-ankle pulse wave velocity and ankle-brachial blood pressure index on mortality in hemodialysis patients. Am J Kidney Dis. 2005; 46: 688–696. 16183424 10.1053/j.ajkd.2005.06.016

[19] Feringa HH Bax JJ van Waning VH Boersma E Elhendy A Schouten O Tangelder MJ van Sambeek MH van den Meiracker AH Poldermans D The long-term prognostic value of the resting and postexercise ankle-brachial index. Arch Intern Med. 2006; 166: 529–535. 16534039 10.1001/archinte.166.5.529

[20] Feringa HH Karagiannis SE Schouten O Vidakovic R van Waning VH Boersma E Welten G Bax JJ Poldermans D Prognostic significance of declining ankle-brachial index values in patients with suspected or known peripheral arterial disease. Eur J Vasc Endovasc Surg. 2007; 34: 206–213. 17481930 10.1016/j.ejvs.2007.02.018

[21] Criqui MH Ninomiya JK Wingard DL Ji M Fronek A Progression of peripheral arterial disease predicts cardiovascular disease morbidity and mortality. J Am Coll Cardiol. 2008; 52: 1736–1742. 19007695 10.1016/j.jacc.2008.07.060 PMC2871035

[22] Norgren L Hiatt WR Dormandy JA Nehler MR Harris KA Fowkes FG Inter-Society Consensus for the Management of Peripheral Arterial Disease (TASC II). J Vasc Surg. 2007; 45 (Suppl): S5-S67. 17223489 10.1016/j.jvs.2006.12.037

[23] Chiu YW Adler S Budoff M Takasu J Ashai J Mehrotra R Prevalence and prognostic significance of renal artery calcification in patients with diabetes and proteinuria. Clin J Am Soc Nephrol. 2010; 5: 2093–2100. 20705966 10.2215/CJN.03730410 PMC3001764

[24] Chiu YW Adler SG Budoff MJ Takasu J Ashai J Mehrotra R Coronary artery calcification and mortality in diabetic patients with proteinuria. Kidney Int. 2010; 77: 1107–1114. 20237457 10.1038/ki.2010.70

[25] Knowler WC Coresh J Elston RC Freedman BI Iyengar SK Kimmel PL Olson JM Plaetke R Sedor JR Seldin MF The Family Investigation of Nephropathy and Diabetes (FIND): design and methods. J Diabetes Complications. 2005; 19: 1–9. 15642484 10.1016/j.jdiacomp.2003.12.007

[26] Levey AS Bosch JP Lewis JB Greene T Rogers N Roth D A more accurate method to estimate glomerular filtration rate from serum creatinine: a new prediction equation. Ann Intern Med. 1999; 130: 461–470. 10075613 10.7326/0003-4819-130-6-199903160-00002

[27] O’Hare AM Glidden DV Fox CS Hsu CY High prevalence of peripheral arterial disease in persons with renal insufficiency: results from the National Health and Nutrition Examination Survey 1999-2000. Circulation. 2004; 109: 320–323. 14732743 10.1161/01.CIR.0000114519.75433.DD

[28] de Vinuesa SG Ortega M Martinez P Goicoechea M Campdera FG Luño J Subclinical peripheral arterial disease in patients with chronic kidney disease: prevalence and related risk factors. Kidney Int Suppl. 2005; 67: S44–S47. 15613068 10.1111/j.1523-1755.2005.09310.x

[29] van Battum P Schaper N Prompers L Apelqvist J Jude E Piaggesi A Bakker K Edmonds M Holstein P Jirkovska A Mauricio D Ragnarson Tennvall G Reike H Spraul M Uccioli L Urbancic V van Acker K van Baal J Ferreira I Huijberts M Differences in minor amputation rate in diabetic foot disease throughout Europe are in part explained by differences in disease severity at presentation. Diabet Med. 2011; 28: 199–205. 21219430 10.1111/j.1464-5491.2010.03192.x

[30] Criqui MH Langer RD Fronek A Feigelson HS Klauber MR McCann TJ Browner D Mortality over a period of 10 years in patients with peripheral arterial disease. N Engl J Med. 1992; 326: 381–386. 1729621 10.1056/NEJM199202063260605

[31] Newman AB Siscovick DS Manolio TA Polak J Fried LP Borhani NO Wolfson SK Ankle-arm index as a marker of atherosclerosis in the Cardiovascular Health Study. Circulation. 1993; 88: 837–845. 8353913 10.1161/01.cir.88.3.837

[32] Leng GC Fowkes FG Lee AJ Dunbar J Housley E Ruckley CV Use of ankle brachial pressure index to predict cardiovascular events and death: a cohort study. BMJ. 1996; 313: 1440–1444. 8973232 10.1136/bmj.313.7070.1440 PMC2352992

[33] Newman AB Tyrrell KS Kuller LH Mortality over four years in SHEP participants with a low ankle-arm index. J Am Geriatr Soc. 1997; 45: 1472–1478. 9400557 10.1111/j.1532-5415.1997.tb03198.x

[34] Brevetti G Schiano V Verdoliva S Silvestro A Sirico G De Maio J Lanero S Chiariello M Peripheral arterial disease and cardiovascular risk in Italy. Results of the Peripheral Arteriopathy and Cardiovascular Events (PACE) study. J Cardiovasc Med (Hagerstown). 2006; 7: 608–613. 16858240 10.2459/01.JCM.0000237909.26377.9f

[35] Diehm C Allenberg JR Pittrow D Mahn M Tepohl G Haberl RL Darius H Burghaus I Trampisch HJ Mortality and vascular morbidity in older adults with asymptomatic versus symptomatic peripheral artery disease. Circulation. 2009; 120: 2053–2061. 19901192 10.1161/CIRCULATIONAHA.109.865600

[36] Itaya H Shiba M Joki N Nakamura M Combined assessment of chronic kidney disease and subclinical peripheral artery disease used to predict future cardiac events. Nephrology (Carlton). 2010; 15: 230–235. 20470284 10.1111/j.1440-1797.2009.01188.x

[37] Schwarz U Buzello M Ritz E Stein G Raabe G Wiest G Mall G Amann K Morphology of coronary atherosclerotic lesions in patients with end-stage renal failure. Nephrol Dial Transplant. 2000; 15: 218–223. 10648668 10.1093/ndt/15.2.218

[38] Tsunoda K Shimajiri Y Morita S Furuta M Kadoya Y Yamada S Nanjo K Sanke T Chronic kidney disease has a more powerful impact on peripheral arterial disease than metabolic syndrome in Japanese type 2 diabetic patients. Metab Syndr Relat Disord. 2009; 7: 323–326. 19558271 10.1089/met.2008.0074

[39] Silvestro A Diehm N Savolainen H Do DD Vögelea J Mahler F Zwicky S Baumgartner I Falsely high ankle-brachial index predicts major amputation in critical limb ischemia. Vasc Med. 2006; 11: 69–74. 16886836 10.1191/1358863x06vm678oa

[40] Mehrotra R Disordered mineral metabolism and vascular calcification in nondialyzed chronic kidney disease patients. J Ren Nutr. 2006; 16: 100–118. 16567266 10.1053/j.jrn.2006.01.006

[41] Kennedy M Solomon C Manolio TA Criqui MH Newman AB Polak JF Burke GL Enright P Cushman M Risk factors for declining ankle-brachial index in men and women 65 years or older: the Cardiovascular Health Study. Arch Intern Med. 2005; 165: 1896–1902. 16157835 10.1001/archinte.165.16.1896

[42] Doobay AV Anand SS Sensitivity and specificity of the ankle-brachial index to predict future cardiovascular outcomes: a systematic review. Arterioscler Thromb Vasc Biol. 2005; 25: 1463–1469. 15879302 10.1161/01.ATV.0000168911.78624.b7

[43] McKenna M Wolfson S Kuller L The ratio of ankle and arm arterial pressure as an independent predictor of mortality. Atherosclerosis. 1991; 87: 119–128. 1854359 10.1016/0021-9150(91)90014-t

[44] Suominen V Uurto I Saarinen J Venermo M Salenius J. PAD as a risk factor for mortality among patients with elevated ABI – a clinical study. European journal of vascular and endovascular surgery: the official journal of the European Society for Vascular Surgery 2010; 39: 316-322. 10.1016/j.ejvs.2009.12.003 20089422

